# The complete chloroplast genome of *Aganope dinghuensis* (Fabaceae)

**DOI:** 10.1080/23802359.2020.1871437

**Published:** 2021-02-09

**Authors:** Zheng-Feng Wang, Qiao-Mei Qin, Hong-Lin Cao

**Affiliations:** a Key Laboratory of Vegetation Restoration and Management of Degraded Ecosystems, South China Botanical Garden, Chinese Academy of Sciences, Guangzhou, China; b Center for Plant Ecology, Core Botanical Gardens, Chinese Academy of Sciences, Guangzhou, China; c Southern Marine Science and Engineering Guangdong Laboratory (Guangzhou), Guangzhou, China; d Guangdong Eco-engineering Polytechnic, Guangzhou, China

**Keywords:** *Aganope dinghuensis*, chloroplast, genome assembly, Pacbio long read sequencing

## Abstract

*Aganope* is a genus in the family Fabaceae, with only 11 species. They are distributed throughout Asia and Africa. *Aganope dinghuensis*, a newly reported species, is native to China with a restricted distribution. We, therefore, report its complete chloroplast genome for better future conservation. The chloroplast genome of *A*. *dinghuensis* is 143,690 bp, with a GC content of 35.32%. In the genome, a pair of inverted repeat regions of 13,015 bp each, a large single-copy region of 98,824 bp, and a small single-copy region of 18,836 bp were identified. Genome annotation identified 115 genes, comprising 74 protein-coding genes, 8 ribosomal RNA genes, and 33 transfer RNA genes. Repeat analysis indicates that the chloroplast genome of *A*. *dinghuensis* contains 126 simple sequence repeats (SSR), of which the majority are A/T mononucleotides. Phylogenetic analysis revealed that *A*. *dinghuensis* is a sister to the clade that includes *Indigofera tinctoria*, *Desmodium uncinatum*, *Sarcodum scandens*, *Wisteria brachybotrys*, and *Callerya nitida*.

*Aganope*, a genus of flowering plants, belongs to the Fabaceae family. Worldwide there are only 11 known *Aganope* species (http://www.plantsoftheworldonline.org/), distributed in Asia and Africa. Biogeographic analysis indicated that *Aganope* originated in the Miocene, and both Africa and Asia are its ancestral areas (Sirichamorn et al. 2014). *Aganope* species are mainly shrubs or small trees, but some are climbing. *Aganope dinghuensis* (P. Y. Chen) T. C. Chen & Pedley is native to China. It is a newly reported species, previously called *Derris dinghuensis* (Chen 1984). It is a liana with a woody and twining stem and grows in forests along rivers or roadsides. The plant contains rotenone, which can be used as an insecticide. Unlike some widely distributed *Aganope* species, such as *A. heptaphylla*, *A*. *dinghuensis* has a restricted distribution in southern China, and only two populations have been found in the wild. It is considered a vulnerable species, and therefore, we report its complete chloroplast genome to provide a germplasm resource for better future conservation.

Fresh leaves of *A*. *dinghuensis* were sampled from Dinghushan, Zhaoqin City, China (23°7′13″N, 112°28′59″E). The voucher specimen was deposited at the Herbarium of South China Botanical Garden (No IBSC 0858303). The genomic DNA of *A*. *dinghuensis* was first extracted by a modified CTAB (cetyltrimethylammonium bromide) method, and the extracted DNA was then sheared to construct a 10 kb library and sequenced using the Pacbio Sequel system. After sequencing, approximate 35 Gb Pacbio long reads were generated. Subsequently, Organelle_PBA 1.0.8 (Soorni et al. 2017) were applied to assemble the chloroplast genome with default parameters. The CPGAVAS2 (Shi et al. 2019) was used to identify genes and simple sequence repeats (SSR) in the assembled genome. The identified genes were validated by tbl2asn v3.10.0-1127.10.1.el7 (https://www.ncbi.nlm.nih.gov/genbank/tbl2asn2/), and the errors were corrected by manual curation versus reference sequences. The genome and its annotated genes were then submitted to GenBank under the accession number MW133234. Phylogenetic analysis for *A*. *dinghuensis* and another 24 species (Figure 1) in the Fabaceae family was performed by mashtree v1.2.0 (Katz et al. 2019) and the reliability of the phylogenetic tree was tested using 1000 bootstrap replications.

**Figure 1.**
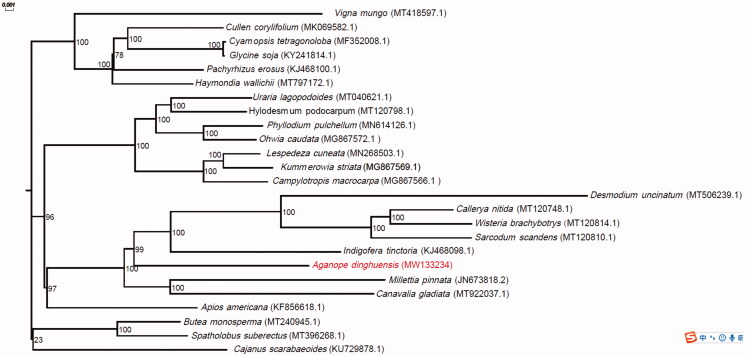
Phylogenetic relationship for *Aganope dinghuensis* and 24 additional species in Fabaceae using their complete chloroplast genomes. The genomes of additional species were download from GenBank and their GenBank accession numbers are shown in parentheses. Bootstrap percentages (1000 replicates) are shown at nodes.

The assembled chloroplast genome of *A*. *dinghuensis* had a circular structure. The length of the genome was 143,690 bp, and the GC content was 35.32%. The genome consisted of a pair of inverted repeat regions (IRA and IRB, each of 13,015 bp) separated by a large single-copy region of 98,824 bp and a small single-copy region of 18,836 bp. Genome annotation revealed a total of 115 genes and 126 SSRs. The genes included 74 protein-coding genes, 8 ribosomal RNA genes, and 33 transfer RNA genes. Among the SSRs, only two types of repeat, mononucleotide and dinucleotide, were detected (Table S1). The most abundant type of repeat was mononucleotide (83.33%) and the majority of the repeat was A/T (77.78%). For dinucleotide repeat only AT/TA was found. Phylogenetic analysis revealed that *A*. *dinghuensis* was a sister to the clade including *Indigofera tinctoria*, *Desmodium uncinatum*, *Sarcodum scandens*, *Wisteria brachybotrys*, and *Callerya nitida* with high bootstrap support (99%; Figure 1).

## Data Availability

The complete chloroplast genome sequences of *Aganope dinghuensis* have been deposited in GenBank under the accession number MW133234 and is also accessible at https://doi.org/10.13140/RG.2.2.34675.35364. The associated BioProject, SRA, and Bio-Sample numbers are PRJNA682008, SRR13179903, and SAMN16976534 respectively.
